# Progress and Challenges in Colorectal Cancer Screening

**DOI:** 10.1155/2012/846985

**Published:** 2012-04-04

**Authors:** Enrique Quintero, Cesare Hassan, Carlo Senore, Yutaka Saito

**Affiliations:** ^1^Gastroenterology Department, Hospital Universitario de Canarias, 38320 La Laguna, Tenerife, Spain; ^2^Gastorenterology Department, Nuovo Regina Margherita Hospital, 00153 Rome, Italy; ^3^CPO Piemonte (Department), AOU S. Giovanni Battista, 10123 Turin, Italy; ^4^Endoscopy Division, National Cancer Center Hospital, Tokyo 104-0045, Japan

## Abstract

Although faecal and endoscopic tests appear to be effective in reducing colorectal cancer incidence and mortality, further technological and organizational advances are expected to improve the performance and acceptability of these tests. Several attempts to improve endoscopic technology have been made in order to improve the detection rate of neoplasia, especially in the proximal colon. Based on the latest evidence on the long-term efficacy of screening tests, new strategies including endoscopic and faecal modalities have also been proposed in order to improve participation and the diagnostic yield of programmatic screening. Overall, several factors in terms of both efficacy and costs of screening strategies, including the high cost of biological therapy for advanced colorectal cancer, are likely to affect the cost-effectiveness of CRC screening in the future.

## 1. Introduction

Colorectal cancer (CRC) can be prevented as more than 85% of tumors arise in a premalignant polyp. Therefore, the aim of CRC screening is to reduce mortality and, if possible, the incidence of the disease by identifying individuals with presymptomatic neoplastic lesions who may require further examination and treatment [1].

The most extended CRC screening strategies are based either on annual or biennial fecal occult blood tests, with colonoscopy reserved for patients testing positive, or on endoscopic procedures performed one-time or every 5 years for flexible sigmoidoscopy (FS) or every 10 years in the case of colonoscopy. In addition, other screening procedures, such as CT colonography and fecal DNA analysis, are under current investigation. 

Compliance to screening and accuracy of the screening tests are the two major determinants of the effectiveness of a screening program. Although evidence from several studies have shown that some of the above-mentioned strategies are effective [1–4] and cost-effective [5], participation is still poor with compliance rates lower than 50% in population-based programs carried out in Europe and USA. This is a current bone of contention since it has been suggested that new strategies, including either the combination of established tests (i.e., fecal immunochemical test (FIT) and endoscopic procedures) or the implementation of novel screening tests that do not require bowel preparation and do not cause discomfort (i.e., blood biomarkers) might substantially improve the current adherence to CRC screening. 

During the last decade there has been a marked improvement in the performance of the screening tests for detecting CRC and preneoplastic lesions. Several randomized controlled trials have shown that the novel semiquantitative fecal immunochemical tests (FITs) are better accepted [6] and have a better performance than the traditional chemical (guaiac) test for detecting colorectal neoplasia [7, 8]. In addition, new technologies (i.e., image-enhanced endoscopy (IEE)) have been introduced to improve endoscopic polyp detection [9], although its influence in the framework of screening still needs to be defined. 

CRC screening is not only an effective tool for reducing CRC-related mortality and incidence, but also has been estimated to do so at acceptable costs. Almost ten years ago, the US Preventive Services task force (USPSTF) estimated that costs per life-year gained of the different screening strategies ranged between 10.000$ and 25.000$. However, several of the screening methods considered today as first-choice screening strategies (i.e., FIT) were not available at the time of the USPSTF report. Recent evidence has confirmed that CRC screening continuous to be cost effective compared to no screening, irrespective of the screening test used. However, cost-effective analysis does not clarify what is the optimal test for CRC screening, because of the uncertainty surrounding many aspects of such interventions.

In this paper, we discuss the possible contribution of the new endoscopic technologies, as a useful tool for screening colonoscopy. In addition, we put in perspective the current strategies for CRC population-based screening and discuss the convenience for combining the established strategies in the near future. Finally, we address the factors that determine cost-effective analysis in the setting of CRC screening.

## 2. Impact of New Endoscopic Technologies in CRC Prevention

Colonoscopy is considered the gold standard for the detection of neoplastic lesions at risk of progression to CRC and is recommended as a first-line screening test in average and high-risk populations. Its main advantage is that removal of adenomas or early cancer can be performed during the same procedure whereas all other screening tests require colonoscopy for confirmation and removal. However, colonoscopy has also important limitations: first, the risk of major complications (postpolypectomy bleeding and perforation [10] are estimated very low (0.1 and 0.3%, resp.) for symptomatic patients, but still may be relevant in the framework of screening programs, where asymptomatic subjects are explored; second, although colonoscopy is considered the reference standard for the detection of colonic neoplasia, polyps are still missed. A substantial adenoma miss rate of 20 to 26% for any adenoma and of 2.1% for large adenomas (≥10 mm) was reported in tandem colonoscopy studies [11]. Adenoma detection rate is highly dependent on quality standards including colonoscopist skills, technology, and several patient-related factors [12]. This section reviews the new endoscopic technologies that may significantly reduce the adenoma miss detection rate associated with conventional colonoscopy.

### 2.1. Wide-Angle Colonoscopy

A prototype wide-angle colonoscope did not eliminate polyp miss rates [13], however, simulation of a colonoscope with a 170-degree field of view resulted in 6% reduction compared with the commonly available 140-degree angle of view of most colonoscopes. The latest generation of wide-angle high-definition colonoscopes improves rates of adenoma detection by 22%, compared with older technology endoscopes used in routine private practice [14].

### 2.2. The Third Eye Retroscope (TER)

The Third Eye Retroscope (TER) (Avantis Medical Systems, Inc, Sunnyvale, CA, USA) is a newly developed imaging device providing a retrograde image of the colon. This device has proved effective in improving polyp detection on the proximal sides of folds in animal models [15] and has also proved safe in humans [16, 17]. Contrarily to wide-angle colonoscopy, TER has the inconvenience of requiring the visualization of dual monitors by one endoscopist and the occupation of the scope's biopsy channel, making difficult its use in routine clinical practice.

### 2.3. Image-Enhanced Endoscopy (IEE)

New image-enhanced endoscopy (IEE) such as narrow band imaging (NBI) system and endoscopic autofluorescence imaging (AFI) system have been developed in order to visualize flat-type colorectal tumor or tumor's surface mucosal or capillary pattern clearly.

#### 2.3.1. Narrow Band Imaging (NBI) System

NBI has been shown to be helpful for early detection of superficial cancer in the head and neck region and the esophagus [18]. In the colorectal region, this modality was expected to enable early detection of adenoma lesions; however, both positive [19, 20] and negative [21–24] results have been reported, and some researchers have concluded no improvement in the adenoma detection rate of NBI compared with white-light colonoscopy (WLC).

One reason for these conflicting findings could be the difference between the optical-electronic technologies employed in videoendoscopes in the NBI systems used sequential system (LUCERA; Olympus Optical Co.) versus nonsequential system (EXERA II; Olympus Optical Co.). Further, differences in the endoscope (low resolution versus high resolution) and imaging (surface structure enhancement and index of hemoglobin color enhancement) settings can influence the detection of the same lesion. Moreover, the colonoscopist's experience may have a considerable impact on the detection rate: if the colonoscopist does not have sufficient training in chromoendoscopy of flat and depressed lesions with an NBI system, the usefulness of NBI for adenoma detection may not be evident. Finally, most of the previous studies used a single-center design.

We reported that the A-5 image setting of the surface structure enhancement function together with the level 3 adaptive index of hemoglobin color enhancement function seem to be the most suitable for detection of colorectal adenomas [25, 26]. To overcome the aforementioned confounding factors, we evaluated the colonic adenoma detection rate of NBI versus WLC by using consistent NBI system, endoscope, and imaging settings in a multicenter randomized trial (RCT) using cross-over design. This RCT was conducted in the right colon for 813 patients using high-definition colonoscopy and NBI settings with surface structure enhancement. This large randomized trial did not show any objective advantage of NBI over WLC in terms of improved neoplastic lesion detection.

#### 2.3.2. Autofluorescence Imaging (AFI) System

AFI produces real-time pseudocolor images to identify gastrointestinal malignancies [27, 28] as well as malignancies of larynx, cervix, lung, and bladder. During AFI colonoscopy, nonneoplastic lesion appears green, while neoplastic lesion has a magenta (reddish purple) image. The usefulness of AFI for differential diagnosis between neoplastic and nonneoplastic lesions has been reported; however, there is scarce information regarding its effectiveness for colorectal polyp detection in comparison with conventional white-light colonoscopy (WLC). Therefore, the utility of AFI to improve colorectal tumor detection still remains controversial. We therefore conducted a pilot study to evaluate whether AFI can detect more colorectal polyps than WLC. Interestingly, our study showed that AFI system improved the detection of right-sided colonic polyps, especially flat and/or diminutive adenomatous lesions compared to conventional WLC.

In the future, further trials should be performed to validate the usefulness by the combined use of AFI and NBI system for CRC screening.

### 2.4. Capsule Endoscopy

Colon capsule endoscopy is a new technique to visualize the colon, originating from small bowel imaging. Van Gossum et al. [29] were the first to evaluate the effectiveness in a prospective setting. In high-risk patients, sensitivity and specificity for detecting polyps ≥6 mm were 64 and 84%, respectively, whereas sensitivity and specificity for advanced adenoma detection were 73% and 79%, respectively. The accuracy of the second-generation colon capsule for adenoma detection was promising, with an estimated higher sensitivity and specificity [30]. Compared with full colonoscopy, the accuracy of colon capsule is considerably lower and an even more extensive bowel cleansing is needed. Capsule endoscopy has not yet been evaluated in an average risk screening population.

In summary, total colonoscopy is accepted as the most accurate method of investigation for the large bowel, however, colonoscopy still may miss lesions responsible for cancer development. Several novel devices have been introduced to find flat or depressed tumors or lesions hidden behind folds in the colon. Although some of these emergent technologies such as wide-angle colonoscopy markedly reduce the rate of missed polyps other more sophisticated devices (TER, NBI, and AFI) needs further evaluation.

## 3. New Strategies for Population-Based CRC Screening

### 3.1. Technology Innovation for Established Strategies

The adoption of guaiac-fecal occult blood test (g-FOBT) for CRC screening is supported by sound experimental evidence from the US and European trials, which demonstrated that regular screening is associated with a reduction in CRC specific mortality and, in the context of the US study using rehydrated g-FOBT, also with a reduction in CRC incidence. Although this method, adopted by several nation-wide programs in Europe (Finland, UK, and France) can be suitable for population screening, it carries several limitations. Indeed, the test processing and analysis are not automated and therefore they are labor intensive and involve subjective visual reading, while it is not possible to adjust its cutoff for Hb concentration. Also g-FOBT is not specific for human Hb and it showed a low sensitivity for CRC and even more so for advanced adenomas.

Technology developments have been subsequently introduced to overcome some of these limitations. A significant enhancement has been achieved by using antibodies specific to human globin to detect human blood present in feces. As with g-FOBT, the presence of blood in a fecal sample can be used as a marker to detect significant neoplasia in otherwise asymptomatic people. The potential advantages of these alternative fecal tests based on immunochemical technology (FIT) include the possibility of an automated processing and analysis, allowing for the possibility to adjust the cut off of Hb concentration, and the increase in the test specificity for human Hb. The immunochemical technique increases as well the test sensitivity, as it allows to detect smaller blood losses. The disadvantages of FIT methods include sample instability, which impose specific organizational constraints, and the cost of the test. Cost is however highly dependent on local and national market conditions, while the automated testing process with high throughput devices can offset the higher cost of the kit.

Two recent large trials conducted among average risk people showed a higher attendance and detection rate of advanced adenomas and CRC of FIT compared to g-FOBT screening [6, 8]. These trials confirmed the findings of previous comparative studies suggesting the superiority of FIT over g-FOBT [7, 31–35]. Based on this evidence, FIT was recently recommended as the test of choice for population screening in the European Guidelines on quality assurance for CRC screening [36]. The availability of an increasing variety of FIT devices on the market will likely require the adoption of standard and explicit criteria for assessing those device characteristics, including ease of use, sample stability and transport requirements, reproducibility of test results, cost of the kit and of processing, which need to be taken into account when selecting the most appropriate kit for a specific program.

### 3.2. Endoscopic Screening: New Evidence and Open Issues

Most countries implementing population-based CRC screening programs have adopted g-FOBT and, more recently, FIT. Only a few pilot projects and two population-based programs in Italy adopted FS as a screening option, while colonoscopy was proposed as primary test for opportunistic screening in the USA, Germany, and Poland. New evidence accumulating over the past few years lead to a change of this scenario, both in terms of evidence and of available strategies

#### 3.2.1. Sigmoidoscopy

Evidence for endoscopic screening was based, until last year, on observational studies, showing a reduction of the risk of CRC or of CRC-related death among people undergoing endoscopy, or on trials comparing sigmoidoscopy with g-FOBT or FIT, showing a substantially higher yield of neoplasia of a single FS examination as compared to a single round of g-FOBT or FIT screening. The findings of the sigmoidoscopy screening trials presenting long-term follow-up results [2, 37] consistently indicated that the endoscopic excision of colorectal adenomas is associated with a reduction of CRC incidence and mortality. The estimated incidence reduction in the intention to treat analysis is 23% at 12 years in the UK trial and 18% at 11 years in the SCORE trial; the reduction is higher among people undergoing screening, as shown in the perprotocol analysis, with a 33% and 31% CRC incidence reduction, respectively. Contrary to previous reports [38] suggesting that FS might not be effective among women, the protective effect of FS screening is the same for men as for women in the two trials.

The substantial and long-lasting reduction of CRC incidence, which is still about 80% lower than expected at 10 years in the distal colon, supports the hypothesis of a plateau of the prevalence of distal adenomas [39].

The adoption of a screening strategy based on the offer of the test once in the lifetime seems therefore justified, although the optimal target age range has not been defined yet. In Italy, FS screening is currently offered to subjects aged 58 or 60 [40], targeting a new birth cohort every year, while the UK pilot will target people in the age range 55 to 59. However, no difference in the protective effect of FS screening could be observed in the UK and Italian trials when comparing people younger than 60 to those aged 60 to 64. As long as it might be inefficient, or not feasible, to target all people aged 55 to 64, the issue of defining a specific age range for screening is relevant to the planning of population based interventions. Pooled analyses of the published, as well of the ongoing trials, might be useful to determine if a narrower age range, than the two age classes considered in the main analyses, can be identified.

The reduction of proximal CRC incidence and mortality was low and nonsignificant, ranging between 3% in the UK trial and 15% in the SCORE trial among those who were screened. The observed difference might be related to the higher referral rate for total colonoscopy in the Italian trial, although the estimated additional yield of proximal advanced neoplasia associated with the strategy adopted in the SCORE trial was negligible. The results of the ongoing trials adopting different referral criteria might offer useful information to assess the impact of less restrictive total colonoscopy referral policies on the risk of proximal CRC. The positive impact of high referral rates might be lower than expected, however, as evidence mounts that colonoscopy may not prevent as many cancers in the right colon as in the left.

#### 3.2.2. Colonoscopy

Several observational studies [41–43] demonstrated the overall effectiveness of colonoscopy for reduction of CRC incidence and mortality, but with a marked variance in effectiveness for proximal and distal cancer. A substantial protective effect could be observed for CRC arising in the distal colon, while no effect was observed in the proximal colon. Only one recent case-control study [43] indicated that colonoscopy might represent an effective tool to prevent proximal CRC, although an effect could be observed only among people older than 60. This finding is consistent with the results of the Italian SCORE3 trial [44] comparing FS and colonoscopy, which showed that the detection rate was higher for total colonoscopy screening compared with FS screening only for people aged 60 or older.

It might be related to the known shift to the right of CRCs with age, but the underlying reasons for the observed difference in colonoscopy performance in the proximal and distal colon are still unclear. Uncertainties are related to the role of biological differences that may limit the potential effectiveness of colonoscopy in the proximal colon. The relative frequency of nonpolypoid lesions, which are harder to identify and remove, tends to be higher in the right colonic segments. The predominant genetic pathways of carcinogenesis may differ between left-sided and right-sided cancers, with a higher frequency of serrated neoplasms in the proximal colon. Also, contrary to the trend observed in the distal colon, no evidence of a plateau for the incidence of proximal adenomas has been reported.

The assessment of the protective effect for proximal CRCs achievable with colonoscopy represents therefore the most relevant outcome of RCTs evaluating effectiveness of colonoscopy screening. Comparative studies should be designed to assess whether the magnitude of the incremental benefit of colonoscopy over FS is sufficient to justify the additional risks and costs of colonoscopy for screening in the population. These studies should also shed light on issues concerning the actual implementation of a screening strategy based on colonoscopy, such as the definition of a target age range as well as, eventually, of a screening interval.

### 3.3. Combined Strategies

Given the limitations of available endoscopy methods, strategies combining FS with FIT represent an option which deserves consideration, as it might ensure an additional benefit over FS alone, in particular for proximal lesions. The potential value of this approach, as a recommended option for CRC screening, is supported by a decision analysis using microsimulation models, included in the latest version of the USPSTF, which showed that a strategy using FS and FIT can have an impact, in terms of life years saved, comparable with that of colonoscopy, assuming a comparable participation to the screening process. Data concerning the actual impact of such strategy are only available from colonoscopy studies estimating the relative contribution of FOBT or FIT and of a surrogate FS. Prospective studies aimed at assessing the performance of such strategy in the context of screening interventions targeting average risk people are lacking. The research question of such studies should be focused on identifying the best way to add FIT to a FS screening program to achieve an increased yield of proximal neoplasms while ensuring high participation rates.

### 3.4. Promoting Participation while Ensuring High Quality of the Screening Process

The USPSTF CRC screening guidelines pointed out that for all screening modalities the effectiveness of screening decreases substantially as adherence to the regimen declines. They further stated that at the individual level adherence to a screening regimen will be more important in life years gained than the particular screening regimen selected.

Availability of different tests represents indeed a new scenario for mass screening programs, as subject's preferences and attitudes will likely influence the uptake level achievable with different strategies. There is a growing body of literature suggesting that subjects targeted for CRC screening have clear preferences for specific methods, determined by test characteristics. Also, gender, education, and age are associated with the uptake, according to the reports from several studies showing higher response rate among women invited to undergo FIT [6] and a higher attendance rates to FS screening among men and more educated or younger people [6].

The heterogeneity of patient's preferences for how to be screened would therefore support the adoption of strategies favoring their implementation as a possible mean to improve participation in CRC screening, but available evidence for this approach is still limited.

 Finally, for screening programs to obtain the optimum result, a high quality of the screening process is needed. Poor quality of the exam has been proposed as one of the possible factors explaining the lack of a protective effect of colonoscopy for proximal CRC. Indeed, inadequate performance of colonoscopy may limit its effectiveness, in particular in the proximal colon, as suggested also by the finding of a higher proportion of interval CRC in the right colon compared to the distal colon. Efforts to improve quality are therefore needed, taking into account that colonoscopy represents not only a potential tool for primary screening, but it is also recommended as a diagnostic tool for people with positive results from different primary screening tests. These same efforts should however be implemented also for all the recommended screening methods. A wide variability in adenoma detection rate has been observed in the context of the trials and programs adopting FS [45–48] and also in the context of FOBT/FIT-based screening quality of laboratory procedures deserves adequate scrutiny.

## 4. Cost Effectiveness of CRC Screening in the 21st Century

The main purpose of cost-effective models is to provide reasonable estimates on the expected efficacy and convenience of health interventions to the policy makers and, more in general, to the whole society. This in turn may be expected to drive selective implementation of new policies to reduce the burden of any disease in terms of morbidity/mortality and/or treatment-related costs. A further advantage of cost-effective models is to allow a transparent comparison of efficacy and effectiveness among different specialties, in order to allow a productive distribution of economic and financial resources among the different fields. For this reason, the main results of cost-effective models may be expected to be incorporated in clinically orientated guidelines, impacting eventually the clinical field.

Cost effectiveness is critical when applied to health interventions directed to the general population, because of the relevance of both efficacy and cost outcomes. This occurs with breast, cervical, as well as with CRC screening, in which all the population included within a predetermined age cutoff is expected to be invited for the screening intervention. Because of the ethical implications implied in inviting asymptomatic subjects, policy makers may be willing to be reassured about the potential efficacy of a population intervention in terms of risk/benefit ratio. Secondly, because of the large number of people to be screened, generally several millions in each country, policy makers need to be aware of the magnitude of absolute costs to be invested and on the convenience of the intervention.

The main drawbacks of cost-effective models is that, despite the apparent firmness of the cost-effectiveness ratios, cost-effective estimates resound of the underlying uncertainty on the main assumptions postulated when developing the simulation process. Such uncertainty may affect the confidence on any cost-effective outputs, reducing its role in the policy-making process. For this reason, simulation models will never replace clinical estimates on the efficacy of health interventions, such as randomized or controlled clinical studies, which, on the other hand, are absolutely necessary to reduce the degree of uncertainty surrounding cost-effective estimates.

When dealing with CRC screening, cost-effective analysis has consistently shown the favorable profile of CRC screening, irrespectively of the adopted strategy. Such favorable cost-effectiveness, as compared with other medical interventions (i.e., breast cancer screening or renal hemo-dialysis), appeared to be strictly related with the possibility to prevent not only CRC mortality, but also CRC incidence. Any reduction of CRC incidence will not only nullify the CRC-related mortality, but it will also lead to substantial saving, because of the exclusion of surgery/chemotherapy costs. However, cost-effective simulations did not reach clear conclusions on the optimal test for CRC screening, because of the uncertainty surrounding several aspects of such interventions. Despite less relevant, there is also persistent uncertainty on the optimal age window for CRC screening, as well as on the intervals after negative screening tests or following polypectomy. For this reason, further evolutions in this field may arise from the acquisition of new information on both the efficacy and costs of the different tests recommended for CRC prevention.

### 4.1. Efficacy

Several sources of uncertainty on the relative efficacy of different CRC screening tests exist in simulation models. First, the natural history of the adenoma-carcinoma sequence has only partially been clarified. For instance, the progression from low- to high-risk polyps, as well as among different size classes (i.e., ≤5, 6–9, ≥10 mm), is poorly known. Similarly, the information on the progression from large polyps to CRC only relies on one old radiological study at high risk of selective/interpretative bias. The natural history of CRC is also incompletely clarified. Despite the stage-specific survival rates are well documented, the sojourn times among the different stages before the diagnosis have only indirectly been estimated. Finally, the relative rate between CRC arising along the adenoma-carcinoma sequence and *de novo* CRC is still largely unknown. Because of this uncertainty, models tend to differ on the estimates on the transition rates among the several steps of the CRC cancerogenesis, eventually leading to uncertainty on the superiority of one test over the other. It is self-evident that models in which the adenoma-carcinoma sequence is simulated to be relatively low will favour infrequent, but highly sensitive tests for polyps, such as endoscopy or radiology, whilst models simulating a more accelerated carcinogenesis will favour more frequent CRC-sensitive tests, such as fecal tests. Secondly, there is uncertainty on the long-term efficacy of several screening tests. Such uncertainty mainly depends on the lack of appropriate trials addressing such issue. For instance, there is uncertainty on the long-term efficacies of immunochemical fecal test in preventing both CRC mortality and incidence. Similarly, there is uncertainty on the additional efficacy when repeating such tests more frequently (annual versus biannual). Despite widely used as opportunistic screening test, colonoscopy, differently from sigmoidoscopy, has never been fully validated in randomized trials. Moreover, there are wide discrepancies on the exact rate of CRC prevention with this technique, ranging from 30% to virtually 100%. Thirdly, there is uncertainty on how selective strategies of prepolypectomy filtering may affect the eventual efficacy of noninvasive strategies. For instance, it is unclear whether the selective exclusion of ≤5 mm or ≤10 mm polyps with CT colonography or capsule endoscopy will sort out in a reduced protection as compared with the endoscopic techniques in which all the polyps are usually removed. Fourthly, when basing simulations on diagnostic rather than population estimates, there is uncertainty on the reproducibility and generalizability of the data. For instance, different immunochemical fecal tests have shown a wide interval of sensitivity values, and, similarly both endoscopic and radiological tests have shown different estimates of accuracy in relation to the study setting.

It is clear that only the progressive acquisition of new and more complete information on all these different aspects related with the efficacy of the tests will allow simulation models to produce a more realistic and valuable comparison among the different tests. For instance, the recent publication of two high-quality trials on sigmoidoscopy substantially reduced the uncertainty on the efficacy of this test on both CRC incidence and mortality, improving the reliability of its modelling. The same will probably applied to colonoscopy, when the final results of the ongoing trials will become available.

### 4.2. Costs

The main determinants of costs in CRC screening modelling are represented by the procedural costs and the costs related with CRC treatment. Procedural costs will in turn depend on the cost estimate and on the actual exploitation of the different procedures. Despite it is relatively simple to obtain realistic estimates of procedural costs under the Medicare scenario, there is a high degree of variability and lack of transparency among the several US insurances used by <65 year old Americans. Similarly, the reimbursement cost for medical procedures in the public health systems in Europe tends to substantially underestimate the actual exploitation of medical and economic resources, artificially improving the cost-effectiveness profile of CRC screening test. Test specificity is also a major determinant of procedural cost. Consequently, the uncertainty on the exact specificity of fecal tests for advanced adenomas or of radiological procedures for the selected polyp cutoff will generate a huge variability on the final cost-effective estimates. A further cause of uncertainty is related with the application of a financial discounting on the costs occurring in the future years, based on the psychological consideration that money to be spent in the present are considered more valuable as compared to money to be spent in the future. Despite understandable, such assumption will tend to underestimate the absolute burden of costs, potentially advantaging more expensive screening strategies, such as endoscopy or radiology. Thirdly, cost-effectiveness ratio tend to mix between the investments needed in the start-up phase with those required in the following workup of the screening program. However, policy makers tend to provide a higher value to the start-up phase, since the required investments will mainly incur in their running period. This may be one of the reasons for which fecal test-screening program are still favoured in most of the European countries.

In conclusion, we may expect a high degree of evolution from cost-effective models. Such evolution should achieve the difficult task to match the progressive acquisition of clinical information with the actual need and availability of the society. The ultimate aim will be to provide a clear ranking of efficacy and costs of the different choices with a minimal degree of uncertainty on the correctness of these estimates.
